# Extraction of Magnetic Field Features to Determine the Degree of Material Strain

**DOI:** 10.3390/ma14061576

**Published:** 2021-03-23

**Authors:** Przemysław Szulim, Szymon Gontarz

**Affiliations:** Institute of Vehicles and Construction Machinery, Warsaw University of Technology, 02-524 Warsaw, Poland; szymon.gontarz@pw.edu.pl

**Keywords:** technical diagnostics, magnetic field, material effort, steel structures, nondestructive diagnostics

## Abstract

Currently, to realize the reliable operation and proper exploitation of complex machines and structures, information regarding the material condition must be obtained. This information should ideally be acquired in a noninvasive manner. In addition, contemporary rapid technological development is conducive to the research and advancement of new methods, including magnetic methods. This publication describes the methods that can enable the extraction of information from the magnetic field, which is valuable for determining the material effort state and performing technical diagnostics. The issue of using the magnetic field to assess the technical condition of structures is a promising trend in technical diagnostics. Moreover, new ways to process the magnetic field information are being identified to connect the observed surface changes in the magnetic field with the significant diagnostic symptoms. This work provides an extensive introduction to the theoretical basis and diagnostic techniques based on measurements of the magnetic field obtained in close proximity to the structure of interest. The key limitations of the method and associated possibilities are highlighted. The model considerations were taken into account to provide a mathematical description of the extraction process and possible interpretations of the acquired signals. According to the received guidelines, the plan and implementation of two experiments are described along with the obtained results, which demonstrated the possibility of identifying valuable information that can be used to determine the state of the material stress and perform diagnostics of steel structures.

## 1. Introduction

Many materials and machines that can present a real risk of a catastrophe due to fatigue wear, exceeding stress limits, or the appearance of plastic deformation have magnetic properties that can affect the local magnetic field, making it possible to increase the variety of nondestructive techniques, which are necessary for the modern management of technical facility operations [1,2,3]. For this reason, along with the technological development of magnetic sensors and measuring instruments, magnetic methods have recently become very attractive. Additionally, bearing in mind the consequences of unforeseen architectural failures and failures of technical facilities, it is necessary to advance science toward the technology of detecting early stages of damage, which is possible thanks to the use of magnetic methods [4,5,6].

In general, magnetic techniques are divided into active and passive techniques. Currently, the group of active magnetic methods is quite large and widely used. The main methods representing this family include the magnetic noise method (Barkhausen method), eddy current method, powder technique, and a method based on magnetic flux leakage. These types of tests require specialized, usually complex, measuring equipment, and the obtained results are sometimes ambiguous. However, these methods enable the detection of the most dangerous defects: flat surface defects and narrow gap defects. Although they play a very important role in technical diagnostics, the methods are adapted to detect existing defects. In parallel with the development of active diagnostic methods, the development of a group of passive diagnostic methods can be observed, which have all the advantages of active methods but, at the same time, do not require the use of artificial sources of magnetic fields, which is associated with the use of complex and expensive devices. The methods that can detect damage in the nucleation phase are the following methods: magnetoacoustic emission (EMA), metal magnetic memory (MMM) method, and other passive techniques [7,8,9,10,11,12,13], which only use the existence of the natural magnetic field of the Earth. These methods are becoming increasingly popular and are the subject of intensive research.

However, due to the variety and complexity of magnetomechanical effects, it is difficult to identify the quantitative measures that allow us to precisely define the diagnostic thresholds for specific magnetic signals. Recently, the most widely used approach is to acquire the magnetic signal by scanning the spatial distribution of the magnetic field of the sample [14] (e.g., MPM). This method uses the phenomenon of memorizing the effects of cyclical and boundary loads on the structure of the material and analyzes the residual magnetic field [15]. Such an assumed phenomena model of an object is very simplified because it does not even take into account changes in magnetic properties, e.g., permeability of the material, with a change in the stress state of the material [16]. Moreover, the current state of the art shows that new models are needed to match the magnetomechanical phenomena [17]. The relationships existing in the field of plastic deformation seem to be particularly problematic because traditional magnetic–stress coupling models are based on the magnetoelastic theory, which cannot explain magnetic phenomena in the plastic deformation range [18]. The results obtained by scientists indicate new possibilities of describing and modeling phenomena, which better describe the observed reality. Along with the new description of physical interactions, new measurement and analysis methods are necessary that will use the emerging possibilities of obtaining information regarding the magnetic signal [19], both in terms of the elastic and plastic deformations. In both these cases, changes in the mechanical properties of the construction material entail changes in the magnetic properties, mainly the magnetization and permeability of the material [20]. The influence of both parameters is observed by the measuring equipment in the magnetic field around the structure but without the possibility of separating them. The possibility of a separate analysis of both factors could significantly contribute to improving the quality of the estimation of the technical condition of the structure.

Regarding this issue, the next chapter indicates potential physical phenomena that are related to the magnetic signal, which may contain useful diagnostic information for the objects under consideration. Next, the article discusses the issue of selecting a sensor with the appropriate sensitivity and the problem of how to observe the magnetic signal, i.e., whether it is to be a measurement at a point, on a certain observation plane, or whether a spatial measurement is needed. These considerations lead to the idea of fusing information from multiple additional sensors, which may prove pivotal in the proposed approach. The next part of the article describes the key stage from the point of view of diagnostics, i.e., finding a magnetic diagnostic parameter that will be sensitive to degradation changes taking place in a technical facility [21,22]. Chapter 3 presents the mathematical basis of the method of information separation from the magnetic field to extract the information that is most closely related to the technical condition of the material. The following chapters describe two experiments and an exemplary interpretation of the results. The first experiment involved flat samples made of 3H13 stainless steel in various stress states. The second involved an analysis of changes in the distribution of the magnetic field on a sample that was carried through successive states of stress until breaking. The proposed methodology, including the process from planning the experiment and selecting the measurement path, through the measurement, to the extraction of diagnostic information, cannot be clearly assigned to either the passive or active magnetic technique. However, it has been shown that the optimal performance of the entire proposed process may lead to the acquisition of qualitatively and quantitatively appropriate magnetic information. By appropriately transforming this information, it was possible to identify specific magnetic, electromagnetic, or magnetomechanical phenomena. The analysis of these phenomena related to the working conditions and the condition of the technical object led to finding an appropriate diagnostic measure that, in accordance with the nature of magnetic methods, described the early stages of the occurring damage.

## 2. Theoretical Background

To utilize the new diagnostics information pertaining to the functional properties of technical objects, we recommend the analytical estimation of the cross-effects, especially those pertaining to the mechanics and magnetics [21,23,24,25,26,27]. Certain researchers indicated that as the stress condition changes in a material with magnetic properties, the material exhibits a magnetic state, which can be shown by the change in the material magnetization intensity. The magnetization intensity can be attributed to the magnetomechanical phenomena, which might be affected by the static or dynamic loads applied to the material specimen and can be appropriately recorded and analyzed if required.

Furthermore, this impact varies depending on the “quantity and quality” of the material that the specific object is made from. Certain bodies can generate a magnetic field around them. Other objects, when placed in an external magnetic field, lead to a distinct change in this field. To model this aspect, the magnetic properties of a ferromagnetic object can be described as a set of magnetic dipoles (**m*_i_***) that are bound together with microcurrents. According to this concept, the magnetic features of a body of a set of dipoles can be characterized by the magnetization vector **M**, which can be defined as a resultant magnetic moment per unit of volume:

(1) $$M=\frac{\sum m_{i}}{\Delta V}.$$

To render the equation transparent and more usable from the diagnostics viewpoint, the following expression can be formulated on the macroscopic scale:

(2) m=f(λ,ω,E,υ,H,T).

*λ* and *ω* are the components of magnetostriction (*λ =* Δ*l/l* and *ω =* Δ*V/V* for linear and volumetric measures, respectively), and *E* and *υ* denote Young’s modulus and Poisson’s ratio, respectively.

When a permanent layout of the magnetic moments occurs, the specimen exhibits external magnetic features (poles). This behavior is typical for permanent magnetic materials, including ferromagnets. In general, ferromagnetic materials include iron, cobalt, nickel, and certain transitional metals pertaining to iron and rare earth elements. When performing diagnostics on engineering structures that are built mainly from steel or cast steel, it is preferable to operate with ferromagnetic soft materials, which usually lose nearly all their magnetization once the magnetizing field is removed and exhibit only residual magnetism, which is considerably smaller than the maximum magnetism. For such materials, the resulting value **M** changes substantially due to an external magnetic field or other physical influences on the object in the presence of such an external field [16].

**Figure 1:**
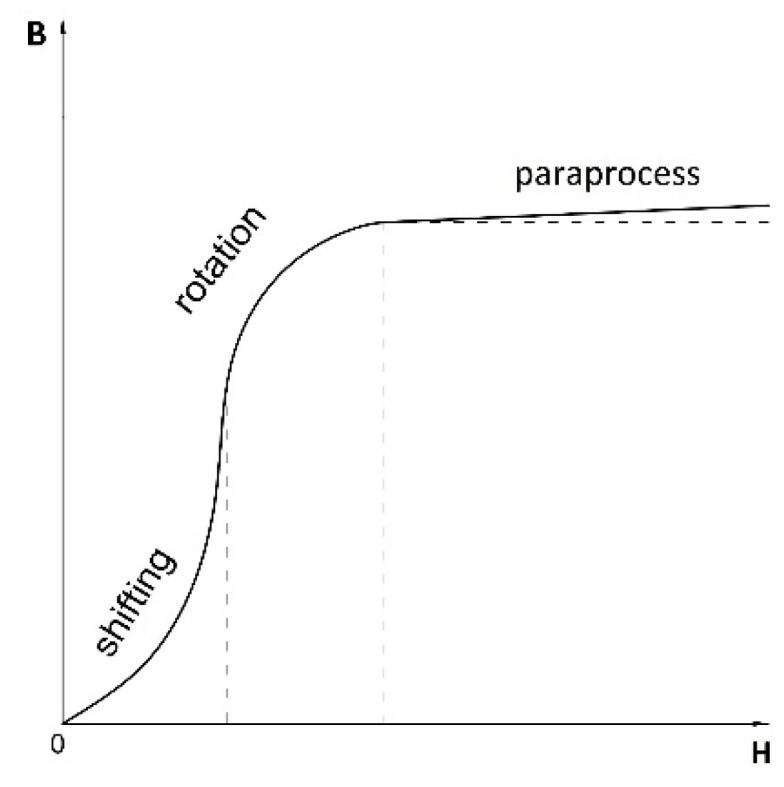
First magnetization curve of ferromagnetic magnetization.

The ferromagnetic behavior can be explained with reference to Weiss’ theory of domains, which indicates that each ferromagnetic material consists of magnetic domains wherein the atoms are arranged in 3D crystal lattices and demonstrate magnetic moments (nonvanishing magnetization vectors). In practical magnetization, the phenomena related to changes in the magnetization state **M**, are highly complex; however, these phenomena can be approximated through a first magnetization curve, as shown in Figure 1.

First, the evolution of the domain structure is reorganized by domain wall displacement. Next, the moment rotation attempts to align the moments of individual areas of spontaneous magnetization parallel to the external field. Finally, the paraprocess occurs, in which after the technical saturation point, as many domains as possible follow the direction of the outer field. A further increase in the magnetic induction occurs with an increase in the **H** field intensity.

The described process refers to an idealized case and is a classic description that is appropriate for the response of a material to the excitation in the form of a change in the magnetic field intensity. However, the actual process is highly complicated because the movements of the walls and rotary movements may overlap, and the nature of the movements is strongly dependent on the structural features of the ferromagnetic material. In this scenario, the specific border points of the domain changes may be ambiguous. In addition, in the presence of other physical influences (see Equation (2)) on the object placed within even a weak magnetic field, the magnetization involving the rotary movement of the domains cannot be excluded, as indicated by the irreversible and nonlinear effects [16].

The described phenomena and behavior of the magnetic material are analogous in the case of magnetization involving magnetomechanical effects, as the mechanical interactions may modify the primary magnetization curve.

According to Equation (2), which presents the magnetization model, and the results of the experiments, a change in the material structure (degradation), stress distribution, material temperature, or magnetic field intensity can influence the magnetization intensity distribution layout **M**(*x*,*y*,*z*) and magnetization intensity distribution of the magnetic induction **B**(*x*,*y*,*z*) in the vicinity of the object being tested. Therefore, the reading of the magnetometer should be understood as an association between the magnetization **M**, the intensity of the external magnetic field **H**, and the magnetic induction **B**, as expressed in the constitutive law equation:

(3) $$B=\mu H=\mu_{0}\left(H+M\right),$$

where *μ* is relative permeability and *μ*_0_ is permeability of free space. The relationship between the magnetization **M**, the intensity of the external magnetic field **H**, and the magnetic induction is a scientifically proven theorem to support nondestructive magnetic testing and monitoring of the structures using active and passive methods.

In general, one can identify three methods of interaction that produce magnetization changes that occur in accordance with the previously described domain processes:

- Stress-affected change via deflection (deflection change in the elastic strain or plastic strain range);
- Change caused by thermal effects (heating the material to a temperature in excess of the Curie point);
- Electromagnetic change (by the action of an external magnetic field).

Electromagnetic magnetization is specific to active methods, while temperature-affected magnetization corresponds to temperatures close to the Curie point. In contrast, in the case of passive methods, the key issue pertains to the stress-affected magnetization.

The stress-affected magnetization procedure in a weak magnetic field (at the level of the natural magnetic field on the Earth’s surface) is essentially different from the technical magnetization procedure conducted under a strong (>>50 A/m) permanent or variable magnetic field, as is currently implemented in non-destructive testing (NDT) inspections. The comprehension of only these variances could provide adequate bases to realize quantitative and qualitative interpretation of the measurement results and enable the fully controlled employment of the observer of the state to diagnose technical objects. In this regard, it is desirable to simplify the numerical model of stress magnetization in a weak magnetic field, which is highly complex compared to that of technical magnetization. The associated division is based on the need to separate both the reversible effects (the Villari effect and its derivatives) and irreversible effects (Δ*E* effect, energy dissipation via Barkhausen noise, change in the magnetization observed after unloading the material, austenite–martensite phase transformation), as indicated in Equations (4)–(6). This division is consistent with the experimentally confirmed mechanism of reversible moving domains and the corresponding irreversible rotation. This division can be expressed in the analytical form as follows:

(4) $$M\left(\sigma ,t\right)=M_{dir}\left(\sigma ,t\right)+M_{irr}\left(\sigma =0,t\right)$$

(5) $$M_{dir}\left(\sigma ,t\right)\to \;\mu_{0}{\left(\frac{\partial M}{\partial \sigma }\right)}_{H}={\left(\frac{\partial B}{\partial \sigma }\right)}_{H}={\left(\frac{\partial \lambda }{\partial H}\right)}_{\sigma }$$

(6) $$M_{irr}\left(\sigma =0,t\right)\to M=\chi H+vH^{2}$$

where σ is stress, t is temperature, v is Rayileigh coefficient, λ is magnetostriction and χ is magnetic susceptibility. The adopted division highlights a certain analogy. The influence of stresses on the magnetic induction produced by an object in a constant external magnetic field can be equated with the change in the magnetostriction under the influence of an external magnetic field at a constant level of stress. However, for the area in which the magnetoplastic phenomenon occurs, the magnetization increases in proportion to the number of cycles of mechanical excitation. With regard to the proposed model, it is advisable to introduce the notion of the “eigenmagnetic field” [16] as a global signal of the magnetic field recorded by the magnetometer. The “eigenmagnetic field” describes the behavior of a magnetoelastically activated material in a weak magnetic field surrounding the material. The concept is broader than that of the diffuse magnetic field because it considers the complex reversible and irreversible effects. This concept allows for the description of a greater number of physical phenomena that more accurately define the state of stress and strain of the material. This description is valuable; however, to obtain this detailed information, the observation method must be extended to include all components of the magnetic field (in the case of self-magnetic flux leakage (SMFL), two components are sufficient) and use appropriate techniques that enable the separation of the effects of interest. The starting point for this concept may be a model description of the reversible/irreversible phenomena, which can be expressed as

(7) $$B_{P}=S\cdot \left(R\cdot B_{E}+B_{M}\right),$$

where the magnetic induction **B_P_** measured in the vicinity of the sample is described. The measurement value includes the component of the terrestrial magnetic induction **B_E_**, and component **B_M_** can be attributed to the permanent magnetization of the sample. The diagonal matrix **S** defines the effect of the closeness of the magnetic material with relative permeability *μ_r_* ≥ 1. Matrix **R** represents the transformations of the local terrestrial field components that are bound in an inertial system to a local magnetic field when observed in a relative system coupled with the tested sample.

A significant limitation to the practical use of this type of model is the complexity of the material structure, access to a limited number of measurements, and the presence of external disturbances. In this regard, the proper recording of the magnetic signals, processing, analysis, and interpretation can enable the enhancement of the capabilities of magnetic diagnostic methods. Consequently, methods that can more accurately estimate the degree of material strain or material degradation are being developed by extracting the information that is not normally available in the measurements. The two proposed methods are discussed in the next section.

## 3. Measurement Model

The idea of separating the measured values is based on the formulation of a measurement model that considers the components of the magnetic field in various reference systems. The first model assumes that the measurement sensor or the sensor matrix is attached to the surface of the tested specimen and that the system is rotated. For this case, we establish two coordinate systems: a local system that is associated with the sensor and sample and a fixed frame of reference. In the case of the latter system, the specimen remains stationary with the sensors, while a slowly changing magnetic flux of low amplitude passes through the sample, causing a change in the field distribution on the sample’s surface.

### 3.1. Measurement Model with Specimen Rotation

The value of the magnetic field in the vicinity of the tested object may consist of many components. The key components pertain to the Earth’s magnetic field and the field resulting from other sources located near the sensor, including residual magnetization (which, among other factors, can be attributed to the stress acting on the tested object). Moreover, the change in the magnetic permeability that occurs as a result of the stresses in the ferromagnetic material leads to local disturbances in the field around the specimen. The magnetic induction **B_b_** at a specific point in space, as measured by the sensor, can be represented as the product of the real induction **B_n_** (in the reference frame) and the rotation matrix $R_{n}^{b}$, thereby allowing for the representation of any vector from the sensor coordinate system to the reference coordinate system:

(8) $$B_{b}=R_{n}^{b}B_{n}.$$

However, the measurement **y^b^** obtained by the sensor is not equal to the **B_b_** field due to the presence of disturbances. All the magnetic field disturbances around the sensor are assumed to be constant in time and space. In the nomenclature related to navigation based on the magnetic field, for which calibration algorithms of magnetometers have been developed [28], the concept of magnetically soft and magnetically hard effects is introduced. Hard magnetic effects manifest as the sources of magnetization in the material of the tested sample. These components contribute to the external magnetic field of the Earth. Moreover, considering the methodology adopted in the experiment, these components are related to the coordinate system of the sensor and are designated as the local component of the $B_{l}^{b}$ field. Soft magnetic effects are related to the magnetic susceptibility of the material near the sensor. These effects manifest as components of the additional field induced by the presence of an external field and contribute to the total field recorded by the sensor. However, these components depend on the direction of the external field and can change not only the size of the measured field but also its direction. This effect is modeled using the matrix **S** (3 × 3). Consequently, the matrix **S**’s coefficients are related to the magnetic permeability of the material. For specimens with no magnetic susceptibility (*μ*_r_ = 1), this matrix reduces to the identity matrix. In the case of samples made of ferromagnetic materials, the matrix coefficients take different values depending on the local changes in the magnetic permeability of the sample, as well as on the shape and dimensions of the sample, as observed in the experiments. Notably, this matrix can be reduced to a diagonal matrix to focus only on the components with the most significant physical interpretation for the given research. The effects of the magnetic field induction are generally nonlinear; however, assuming a weak forcing field (at the level of the Earth’s magnetic field), a linear relationship **B**(**H**) can be assumed:

(9) $$y^{b}=SB_{b}+B_{l}^{b},$$

where:

$$y^{b}=\left[\begin{array}{c} y_{x}^{b}\\ y_{y}^{b}\\ y_{z}^{b} \end{array}\right],\;S=\left[\begin{array}{ccc} S_{x} & 0 & 0\\ 0 & S_{y} & 0\\ 0 & 0 & S_{z} \end{array}\right],\;B_{l}^{b}=\left[\begin{array}{c} B_{l}{}_{x}^{b}\\ B_{l}{}_{y}^{b}\\ B_{l}{}_{z}^{b} \end{array}\right].$$

Under the assumption that the magnitude of the local magnetic field is constant (the magnetic field around the sample is homogeneous), the abovementioned equations can be transformed to a form that can be used to estimate the unknown components of the diagonal matrix **S** and vector $B_{l}^{b}$:

(10) $$\left|S^{-1}\left(y^{b}-B_{l}^{b}\right)\right|=\left|R_{n}^{b}B_{n}\right|=\mathrm{const}.$$

This matrix equation can be simplified to a single nonlinear scalar equation:

(11) $$\frac{{y_{x}^{b}}^{2}}{S_{x}^{2}}+\frac{{y_{y}^{b}}^{2}}{S_{y}^{2}}+\frac{{y_{z}^{b}}^{2}}{S_{z}^{2}}-\frac{2y_{x}^{b}{B_{l}}_{x}^{b}}{S_{x}^{2}}-\frac{2y_{y}^{b}{B_{l}}_{y}^{b}}{S_{y}^{2}}-\frac{2y_{z}^{b}{B_{l}}_{z}^{b}}{S_{z}^{2}}+\frac{B_{l}{{}_{x}^{b}}^{2}}{S_{x}^{2}}+\frac{B_{l}{{}_{y}^{b}}^{2}}{S_{y}^{2}}+\frac{{{B_{l}}_{z}^{b}}^{2}}{S_{z}^{2}}={\left|R_{n}^{b}B_{n}\right|}^{2}=c.$$

This equation can be converted to a form that clarifies the known components and unknown factors *a*_1_–*a*_7_:

(12) $$y_{x}^{b}{}^{2}a_{1}+y_{y}^{b}{}^{2}a_{2}+y_{z}^{b}{}^{2}a_{3}-y_{x}^{b}a_{4}-y_{y}^{b}a_{5}-y_{z}^{b}a_{6}+a_{7}=c,$$

where:

$$a_{1}=\frac{1}{S_{x}^{2}},\;a_{2}=\frac{1}{S_{y}^{2}},\;a_{3}=\frac{1}{S_{z}^{2}},\;a_{4}=\frac{2B_{l}{}_{x}^{b}}{S_{x}^{2}},\;a_{5}=\frac{2B_{l}{}_{y}^{b}}{S_{y}^{2}},\;a_{6}=\frac{2B_{l}{}_{z}^{b}}{S_{z}^{2}},\;a_{7}=\frac{B_{l}{{}_{x}^{b}}^{2}}{S_{x}^{2}}+\frac{B_{l}{{}_{y}^{b}}^{2}}{S_{y}^{2}}+\frac{B_{l}{{}_{z}^{b}}^{2}}{S_{z}^{2}}.$$

For each experiment, many (*n*) measurements of $y_{x}^{b}$, $y_{y}^{b}$, and $y_{z}^{b}$ were obtained to build a matrix equation based on the above equation.

(13) $$\left[\begin{array}{c} \begin{array}{ccc} y_{x_{1}}^{b}{}^{2} & y_{y_{1}}^{b}{}^{2} & \begin{array}{ccc} y_{z_{1}}^{b}{}^{2} & -y_{x_{1}}^{b} & -\begin{array}{ccc} y_{y_{1}}^{b} & -y_{z_{1}}^{b} & 1 \end{array} \end{array} \end{array}\\ \vdots \\ \begin{array}{ccc} y_{x_{n}}^{b}{}^{2} & y_{y_{n}}^{b}{}^{2} & \begin{array}{ccc} y_{z_{n}}^{b}{}^{2} & -y_{x_{n}}^{b} & -\begin{array}{ccc} y_{y_{n}}^{b} & -y_{z_{n}}^{b} & 1 \end{array} \end{array} \end{array} \end{array}\right]\left[\begin{array}{c} a_{1}\\ a_{2}\\ \begin{array}{c} a_{3}\\ a_{4}\\ \begin{array}{c} a_{5}\\ a_{6}\\ a_{7} \end{array} \end{array} \end{array}\right]=\left[c\right]$$

The search for the coefficients of **S** and $B_{l}^{b}$ was performed in two stages. First, certain unknown coefficients were determined using the linear regression method, and these results were considered to be the starting point to identify the remaining coefficients using Newton’s method.

### 3.2. Measurement Model with Excitation

The experiment with sample rotation can only be performed when the tested object is small. To enable the application of the proposed method to large objects, a reverse situation was engineered by creating an artificial magnetic field with the magnitude of the Earth’s field rotating in any direction. This approach enabled the continuous observation of changes in the magnetic properties of the sample material during the stress experiment, which helped to further clarify the phenomena occurring in the material. In the first approach, to reduce the complexity of the stand, the sample was simulated with an artificial field created by the yoke, thereby limiting the range of changes in the magnetic field to one plane. Sinusoidal changes in the excitation were assumed. Due to the assumed low value of the generated flux and current control in the excitation circuit, this assumption could be considered reasonable. In such cases, the local value of the magnetic field on the sample surface can be described through a simple relationship:

(14) $$B_{Mi}=S_{i}\sin\left(\omega t+\varphi_{i}\right)+B_{Ci},$$

where *i* indicates the *x*-, *y*-, or *z*-direction relative to the sample, and *ω* is the frequency of the current excitation

The coefficient *S_i_* is related to the magnetic permeability of the sample and describes the degree of scattering of the excitation magnetic field on the sample’s surface. When *S_i_* = 0, the entire forcing flux is confined to the sample volume and does not leak. The *φ_i_* coefficient is related to the presence of the phase shift between the excitation and registered field, while the *B_C**i**_* component represents the total influence of the sample’s own magnetic induction and the surrounding field. In this scenario, the fields cannot be separated

Both models were designed to extract parameters that could effectively describe the target characteristics of the material for diagnostic purposes. These models were employed in various types of experiments, as described in the subsequent section.

## 4. Test Stand Description

A detailed description of the test stand and the conditions under which the experiment was conducted for the first variant can be found in the literature [8]. However, in the present study, we primarily focused on the experiment pertaining to the novel variant, which involved the model with a certain stimulation.

### 4.1. Stand for the Tests in the Nonstationary Variant (Measurement with Sample Rotation)

**Figure 2:**
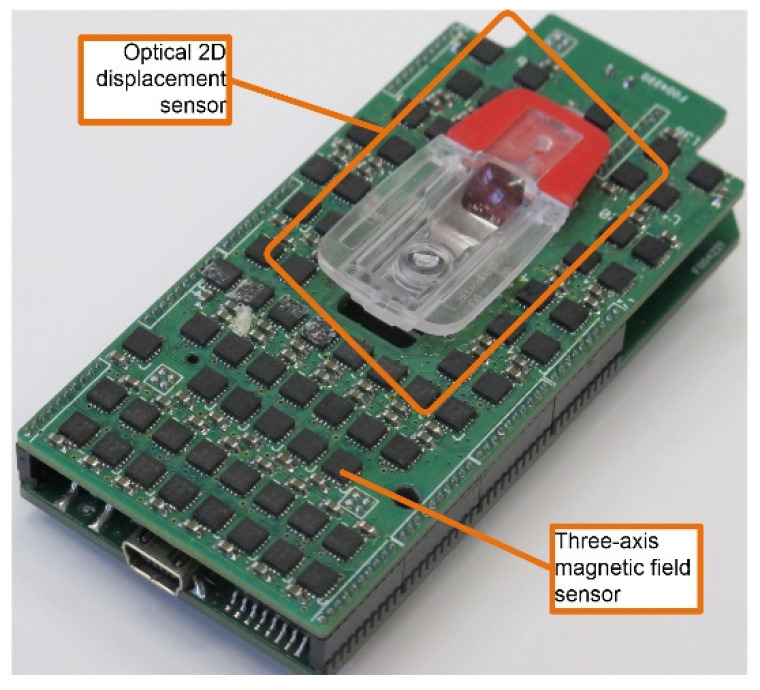
Image of the MagMouse measurement device [8].

The common feature of both experiments was the use of a customized measurement device (MagMouse). The device, as shown in Figure 2, consisted of a matrix of three-axis magnetoresistive sensors, an optical displacement sensor (not used in the experiments), and a microcontroller to realize communication with an external data recording application. Honeywell HMC5983 magnetic sensors (Honeywell, Charlotte, NC, USA) were used to construct the device. The HMC sensor is a digital sensor that converts analog field information from a triad of anisotropic magnetoresistance (AMR) sensors into the digital form. It is possible to program the sampling frequency of the internal analog/digital (A/D) converters and control the gain of the analog signal, thereby enabling variations in the sensitivity and measurement range of the sensor. During the tests, the measurement acquisition frequency and range were set to 75 Hz and 0.8 mT, respectively. In the first variant, the device was rigidly connected to the test sample and rotated in various configurations while recording the field values.

**Figure 3:**
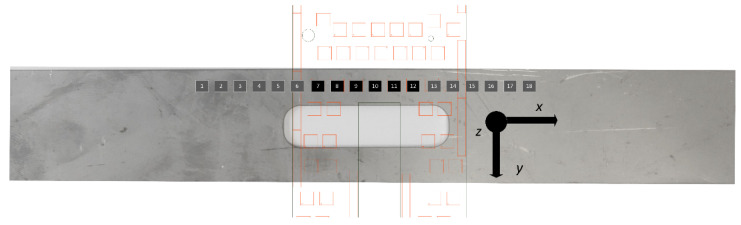
Arrangement of the magnetic field sensors (measurement points) relative to the tested sample.

To enable coverage along the entire length of the sample, the experiment was performed several times by shifting the matrix of the sensors in relation to the tested sample in specific intervals after each experiment. The measurement points on the sample (magnetic field measurement points subject to further evaluation) are illustrated in Figure 3. During the research, data from only one row of matrix sensors were used.

**Figure 4:**
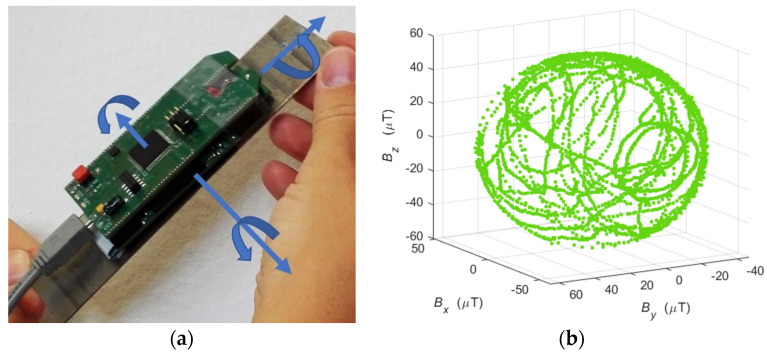
Sample rotation procedure with the sensor matrix (**a**) sample result of the recorded magnetic field creating a characteristic sphere for one selected sensor from the matrix (**b**).

The experimental stage is presented in Figure 4.

After the series of rotations was performed, a magnetic field data set was obtained for each sensor in the matrix. The visualization of the registered field for one measurement point is presented in Figure 4b. A characteristic surface resembling a sphere was created. Next, the set of parameters was extracted according to the model from the first variant. In each experiment, several thousand samples of the magnetic field induction vector were recorded for each measurement point. After estimating the six searched parameters, the effect of filtering out information from noise was obtained. The influence of the sensor measurement noise was significantly reduced. Many series of tests were conducted for specimens in various states of degradation: elastic deformation, plastic deformation, and rupture. The sample specimens used in both variants were made of 3H13/(1.4028 according to EN 10027-2 [29]) steel. The results of the experiment are presented in the subsequent section.

### 4.2. Stand for the Tests in a Stationary Variant (with a Stationary Sample)

**Figure 5:**
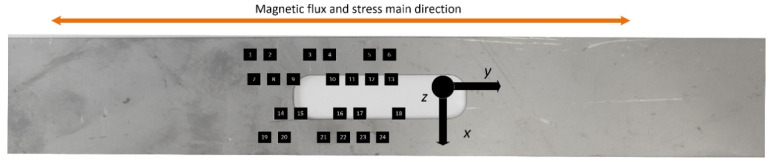
Arrangement of the magnetic field sensors of the matrix relative to the tested sample.

To enhance the knowledge of the processes occurring in the sample during the degradation of the technical condition, and to simultaneously extract the various parameters from the magnetic field signal, the previous variant of the experiment was adopted. The test sample was placed in a testing machine. A yoke forcing a controlled magnetic flux and an array of sensors to measure the field distribution on the surface were placed on the sample. The specimen model, along with the arrangement of the sensors of the magnetic field of the matrix relative to the tested sample, is shown in Figure 5.

**Figure 6:**
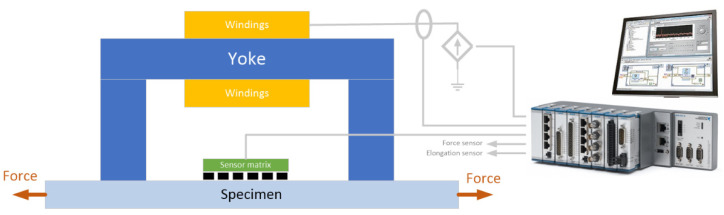
Diagram of the test stand for the second variant of the experiment.

The excitation level was experimentally selected to ensure that the range of field changes was similar to that in the experiment in the first variant. A controlled current source was used to set the current in the yoke. The registration process and signal generation were managed using an application written in the LabView environment. The scheme of the test stand is illustrated in Figure 6.

**Figure 7:**
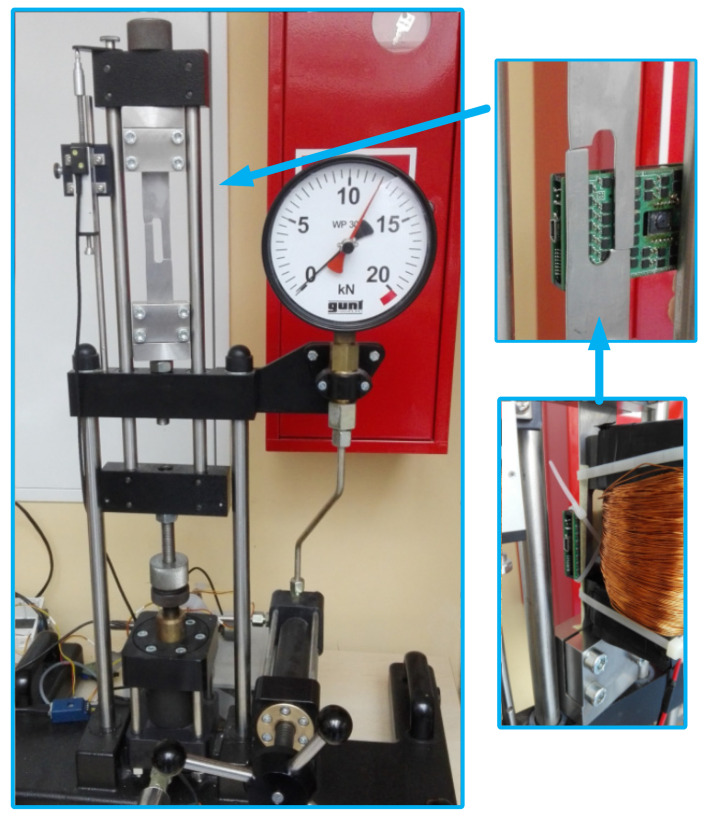
Mutual positions of the yoke, sample, and sensor matrix during the experiment.

Signals, such as those of the yoke current, tensile force, sample elongation, and magnetic field, were recorded. The experiment was performed on samples made of 4H13 steel (1.4034 according to EN 10027-2 [29]). The experiment involved several steps. At each step, a specific value of the excitation force was applied and excitation based on the magnetic flux was generated. Subsequently, the measurement signals were recorded for four periods of the excitation signal. The frequency of the forcing signal was set to be a constant value of 0.05 Hz. The magnetic field sampling frequency was limited by the capabilities of the magnetic field sensors and set to 75 Hz. The yoke, sample, and matrix during the experiment on the testing machine are shown in Figure 7.

## 5. Test Stand Investigation

The results of the tests for both variants of the experiment are presented in this section. The results were analyzed in accordance with the assumed models (Section 3). Consequently, we could extract the parameters describing the behavior of the magnetic field, which changed depending on the material effort state.

### 5.1. Experiment in the Nonstationary Variant (Measurement with Sample Rotation)

**Figure 8:**
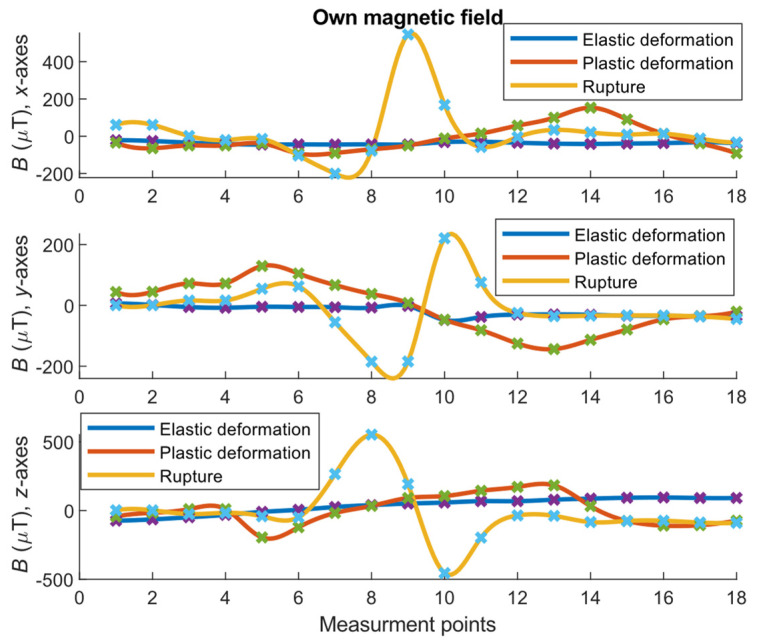
Reversible and irreversible effects associated with different states of the sample strain at successive points on the specimen’s surface (measurement points). The *x*, *y*, and *z* charts correspond to measurements from the *x*-, *y*-, and *z*-axes of magnetic sensors, respectively. The *x*-axis direction was tangential to the main axis of the sample, and the *z*-direction was normal to the sample surface.

**Figure 9:**
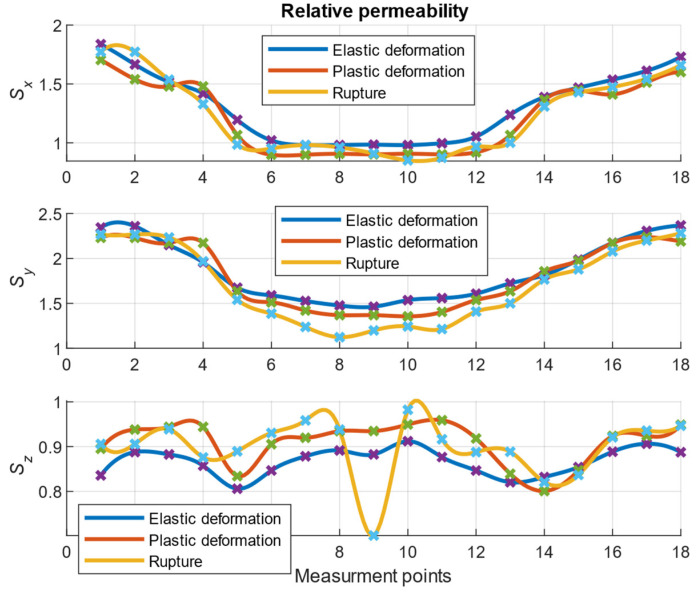
Summary of the changes in the parameter related to the magnetic permeability of the specimen for each axis and different states of stress at successive points on the specimen’s surface (measurement points).

Figure 8 and Figure 9 present the results obtained for the experiment conducted on three specimens with different degradation states. The results pertain to the separated components of the sample’s magnetic field related to the magnetization of the ferromagnetic material.

Figure 8 illustrates the various quantitative and qualitative changes in the magnetic field, which varied with the degradation of the steel material. In the case of parameter **B**, the largest changes occurred in the case of the damaged sample. However, under elastic and plastic deformation, the situation may not be clear. In this scenario, the **S** parameter could provide additional information to recognize the strain states (Figure 9).

Figure 9 shows the influence of the parameter **S** for different effort states of the material. The graph shapes for the *x*- and *y*-axes indicate the changes in the shape of the sample, the width of which was between measurement points 6 and 12. The changes pertaining to the *z*-axis were smaller and may have been related to the constant thickness of the sample. The estimated parameter thus clarified the sensitivity to the shape of the sample. A gradual quantitative change in **S** with the degradation level of the specimen (in all directions) was observed. This observation can help to identify the instant at which the material became plasticized. In raw measurements of the magnetic field, many phenomena affect the result such that degradation estimation is challenging. Using the proposed separation technique, the degradation could be distinguished. Notably, on the axis perpendicular to the sample surface (i.e., the *z*-axis), a larger change occurred in the parameter value for the broken specimen near the material discontinuity. This result was consistent with subsequent tests that demonstrated the significant influence of the changes in the permeability of the material for the range of plastic strains.

Although the described variant (with sample rotation) is not practical in the real world, it proved that even small changes in the structure of the material when interacting with the Earth’s magnetic field yielded a characteristic signal in the form of a magnetic field near the sample. This aspect could be identified using the proposed separation for the parameters **B** and **S**. The analyzed samples differed significantly in terms of the state of deformation; therefore, the magnetic effects related to the effort of the material were significant and easy to register. When less force was applied to the sample and the measurement cannot be repeated, a new approach is required to observe similar diagnostic symptoms in the magnetic field. Such an approach may also require highly sensitive apparatus and special processing. The consideration of real-world issues based on the spatial measurement, including additional information regarding the location, can be useful from a diagnostic viewpoint.

### 5.2. Experiment in the Stationary Variant (with a Stationary Sample)

**Figure 10:**
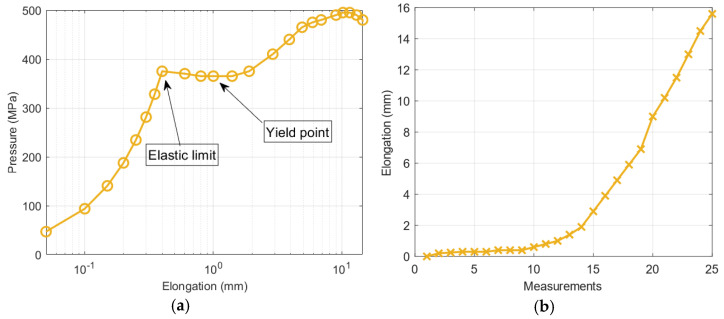
Strength characteristics for the tested specimen (**a**) and changes in the elongation for subsequent measurements (**b**).

Considering the noteworthy results obtained in the first experiment, the authors tested samples pertaining to various states of stress. The strength characteristics for this experiment are described in Figure 10, which clarifies the range of elastic and plastic deformations. Additionally, the characteristic points are marked and were further analyzed. Due to the high dynamics of the changes in various parameters for different states of material stress, we compared these changes as a function of the subsequent measurements and not force.

**Figure 11:**
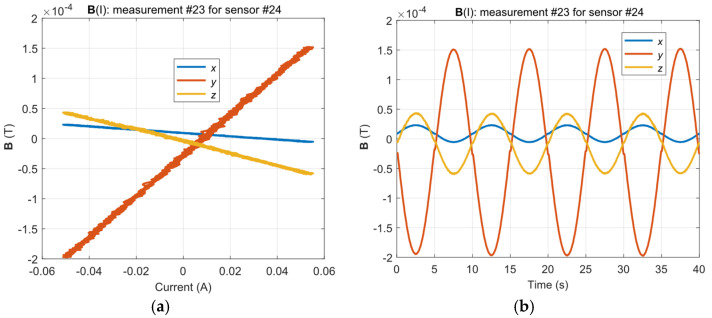
Influence of the alternating excitation current on the measurement of the magnetic field for the selected sensor and measurement (**a**) and the corresponding time plot of the magnetic field (**b**).

The artificial field forced by the yoke flux was selected to match the measurement range of the sensors and to ensure that the work was in the Rayleigh region for which the nonlinearities of the **B**(**H**) characteristic are not clear. Figure 11a shows the **B**(I) graphs for a sample sensor and Figure 11b shows the corresponding time graphs of the field recorded in a single measurement (for a specific material intensity state).

**Figure 12:**
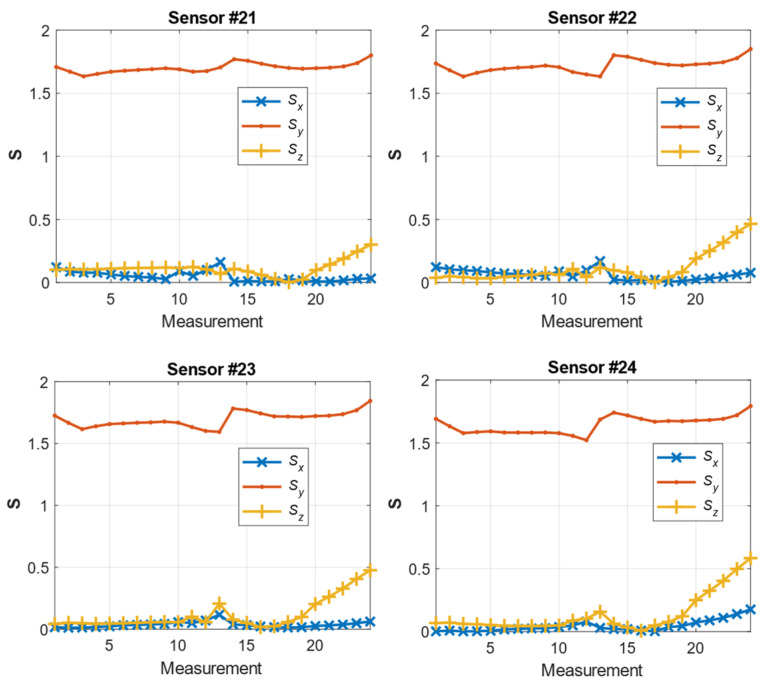
Estimates of the **S** coefficients for successive stress states for sensors 21–24.

**Figure 13:**
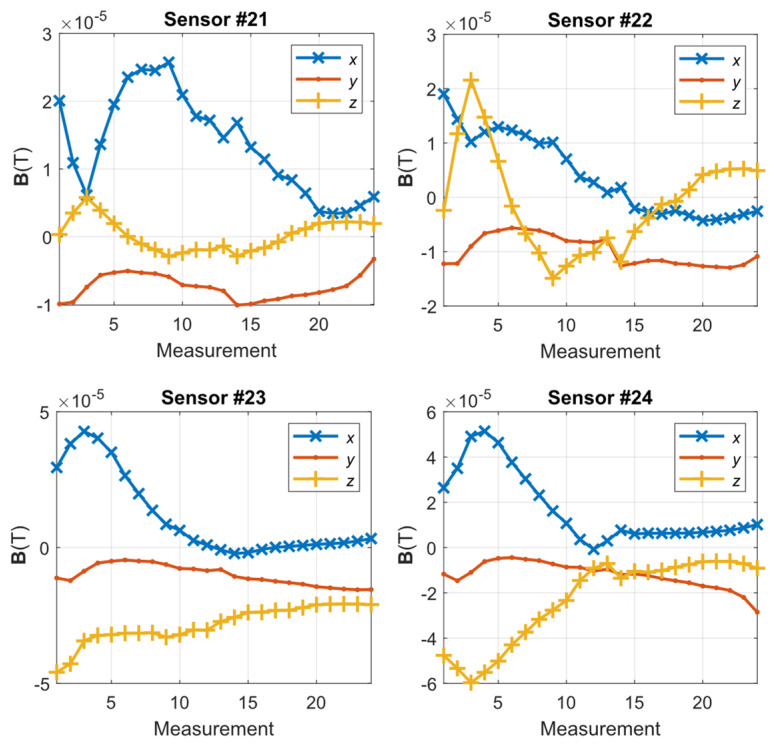
The *x*-, *y*-, and *z*-direction magnetic induction for sensors 21–24 for the specimen discontinuity area.

Figure 12 shows the waveforms of the **S** parameter estimation for the three directions. The highest values pertained to the *y*-direction, as expected, because this direction was the main direction of the magnetic flux of the yoke. In areas 13–15 of the measurement, a characteristic jump occurred near the yield point of the strength characteristic (Figure 10a), i.e., in the range of plastic deformation. Moreover, the *y*-direction changes in this parameter were sustained in the subsequent steps of the experiment; however, for the *x*- and *z*-directions, the changes occurred only in the region close to the point. The estimation of the **S** parameter in the *x*- and *z*-directions highlighted the occurrence of linear changes in the value to promote further plasticization of the material. The *z*-direction appeared to clearly reflect the relationship between the plasticization stage and the change in the material permeability. However, this aspect must be further examined in terms of the influence of the direction of the exciting magnetic field on the estimation of the **S** parameter. Figure 13 shows the estimation of the magnetic field *B_Ci_* recorded by sensor 24. As shown in Figure 5, this sensor was located in the middle of the right arm of the specimen. The range of elastic loads corresponded to measurements 1–8 (according to Figure 10a). The measurement at point 8 was assumed to be the elastic limit. In this area, the most significant changes in the magnetic field vector occurred. The maximum magnitude of the vector corresponded to half the elastic range (approximately 170 MPa) of the specimen’s material. Moreover, the maximum changes in the magnetic field occurred in the *x*-direction (perpendicular to the strain) and the *z*-direction (normal to the specimen surface).

**Figure 14:**
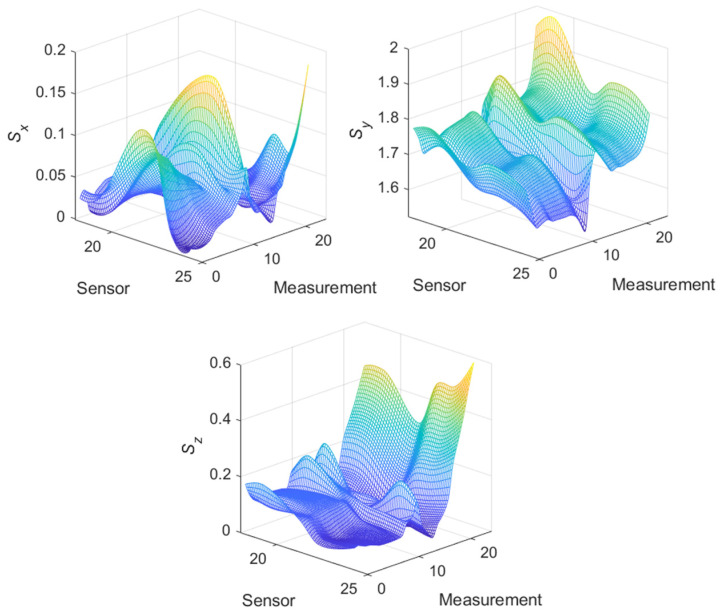
Distribution of the **S** parameter in three directions along the arm for successive states of the material stress.

The measurements presented in Figure 12 and Figure 13 indicate the changes in the estimated parameters pertaining to the characteristic point of the samples. To clarify the spatial distributions of the parameters, the maps for one row of sensors (19–24) are presented. The selected row corresponded to the region of the specimen arm that was subjected to the highest deformation. Figure 14 shows the maps representing the spatial distribution of the **S** parameter for successive measurements.

For the single waveform that is shown in Figure 12 and the remaining measurements on the specimen arm, a characteristic flat fragment of the graph for the elastic deformations was observed. Moreover, beyond the yield point, in the vicinity of measurements 13–15, a characteristic ridge appeared, and a significant increase in the field dispersion occurred, especially in the normal (*z*) direction and the direction perpendicular to the stress (*x*).

**Figure 15:**
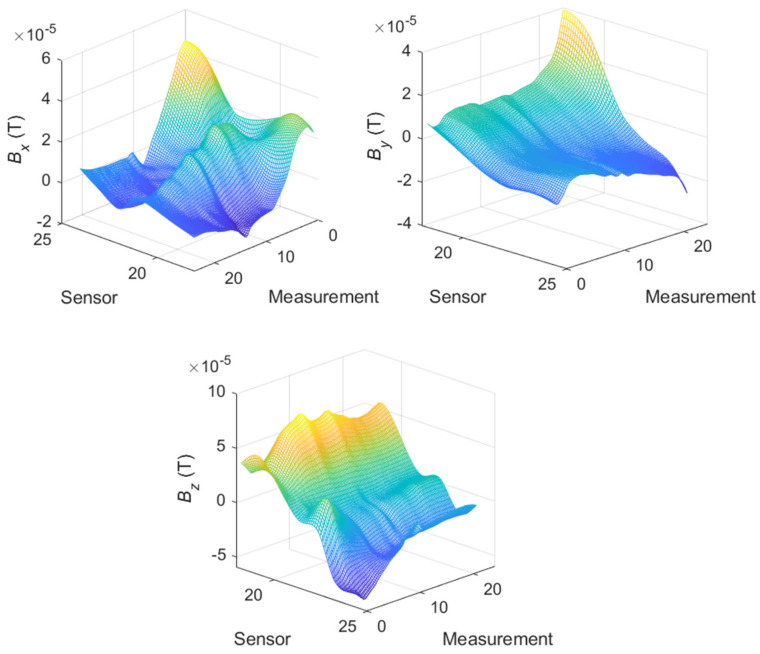
Distribution of the **B** magnetic induction for the broken arm of the specimen for successive states of the material’s stress.

Figure 15 shows the maps representing the spatial distribution of the magnetic flux for successive measurements. Similar to Figure 13, the greatest spatial changes in the field distribution occurred for the elastic region (measurements 1–9), especially in the case of the *x*-direction. These changes were also visible in the plastic area, although the changes were smaller. The polarization of the magnetic pole around the fracture area increased, even in the area of plastic deformation, which was most clearly visible in the measurements pertaining to the *y*-axis. However, the values of the field change for the *y*-axis were more than twice as small as those for the *z*-component (normal) of the magnetic field. The sample broke in areas 20 and 21 of the sensor, in which the field intensity was approximately zero.

The spatial analysis of the degradation of steel elements is highly complex. However, the experiments highlighted several aspects to be considered to clarify the characteristic behaviors, such as an increase in the stress, plasticization, and material rupture.

## 6. Summary

The tests conducted on steel samples showed that an area of stress concentration caused local magnetization of the material, in accordance with the theoretical assumptions. This phenomenon can be used to identify the stress state of a material in the elastic region. However, in the plastic state, the spatial fluctuations of the field were considerably smaller and exhibited different characteristics. These results could be obtained using the proprietary model approach and the conduction of an experiment appropriate for this model. However, the proposed technique involved certain practical limitations due to the required movement of the examined object in relation to the external magnetic field. Therefore, a second experiment was performed, in which stimulation through a modeled external weak magnetic field was incorporated while the sample remained in place. The results for the simplified experiment indicated that this scenario did not disrupt the effects observed in the first experiment. However, in the field of plastic deformation, changes in the field dispersion parameter occurred, which were considerably smaller in the elastic region. Using the proposed method of information extraction, two pieces of information from the eigenmagnetic field signal of an object could be isolated, which reflected the different effects occurring in different states of the material strain. The information obtained from the magnetic field without separation corresponded to a considerably challenging interpretation of the obtained image of the magnetic field distribution. Both effects related to the sample’s inherent magnetization and change in the magnetic permeability affected the resultant distribution of the magnetic field (eigenmagnetic field), and consequently, the measurement of the recorded magnetic field. The boundary of the passage through the two characteristic areas of material strain was very difficult to detect. In addition, the analysis of the individual components of the magnetic field in the described tests enabled the realization of a more accurate inference regarding the direction of stress (the preferred direction of magnetization). Both types of estimated parameters, specifically, the sample’s inherent magnetic field and changes in the permeability, indicated a strong correlation between the directions of the recorded field and force. Therefore, the extension of the experiment from the variant with stationary sample II while controlling the spinning of the forcing field in three planes facilitated the understanding of the relationships between the estimated parameters and state of stress in the sample material.

This presented research did not include cyclic loading of the material. Although such an experiment would be interesting, it is possible to conclude that our magnetic parameters will still be usable and show characteristic behaviors. According to Figures 6 and 7 in [16], in the elastic strain area, some irreversible effects could appear. The proposed diagnostic magnetic parameters should agree with this effect in a quantitative manner that is proportional to the share of reversible and irreversible effects. In the same situation, one can predict the plastic range [30], where the quantitative changes will only influence the proposed parameters in the range of values considered in the present paper. On the other hand, the benefit of extracting magnetic field features should be emphasized in the availability of diagnostic information in the face of low-energy changes or disturbances. Nevertheless, it would be worth doing special research on this topic. Finally, we concluded that the observation of both parameters can be realized to enable a more reliable assessment of the stress state of the material. During the analyses, a characteristic jump of the **S** parameter was observed at Yield point (Figure 10a) of the material strength curve, which was related to the change in the permeability of the sample. This behavior is interesting and the resulting phenomena will be examined in future work.

## Data Availability

Data available on request. The data presented in this study are available on request from the corresponding author.
